# Low-density lipoprotein concentration in the normal left coronary artery tree

**DOI:** 10.1186/1475-925X-7-26

**Published:** 2008-10-17

**Authors:** Johannes V Soulis, George D Giannoglou, Vassilios Papaioannou, George E Parcharidis, George E Louridas

**Affiliations:** 1 Fluid Mechanics Division, School of Engineering, Democrition University of Thrace, Xanthi 67100, Greece; 2 Cardiovascular Engineering and Atherosclerosis Laboratory, 1st Cardiology Department, AHEPA University Hospital, Medical School, Aristotle University of Thessaloniki, Thessaloniki 54631, Greece

## Abstract

**Background:**

The blood flow and transportation of molecules in the cardiovascular system plays a crucial role in the genesis and progression of atherosclerosis. This computational study elucidates the Low Density Lipoprotein (LDL) site concentration in the entire normal human 3D tree of the LCA.

**Methods:**

A 3D geometry model of the normal human LCA tree is constructed. Angiographic data used for geometry construction correspond to end-diastole. The resulted model includes the LMCA, LAD, LCxA and their main branches. The numerical simulation couples the flow equations with the transport equation applying realistic boundary conditions at the wall.

**Results:**

High concentration of LDL values appears at bifurcation opposite to the flow dividers in the proximal regions of the Left Coronary Artery (LCA) tree, where atherosclerosis frequently occurs. The area-averaged normalized luminal surface LDL concentrations over the entire LCA tree are, 1.0348, 1.054 and 1.23, for the low, median and high water infiltration velocities, respectively. For the high, median and low molecular diffusivities, the peak values of the normalized LDL luminal surface concentration at the LMCA bifurcation reach 1.065, 1.080 and 1.205, respectively. LCA tree walls are exposed to a cholesterolemic environment although the applied mass and flow conditions refer to normal human geometry and normal mass-flow conditions.

**Conclusion:**

The relationship between WSS and luminal surface concentration of LDL indicates that LDL is elevated at locations where WSS is low. Concave sides of the LCA tree exhibit higher concentration of LDL than the convex sides. Decreased molecular diffusivity increases the LDL concentration. Increased water infiltration velocity increases the LDL concentration. The regional area of high luminal surface concentration is increased with increasing water infiltration velocity. Regions of high LDL luminal surface concentration do not necessarily co-locate to the sites of lowest WSS. The degree of elevation in luminal surface LDL concentration is mostly affected from the water infiltration velocity at the vessel wall. The paths of the velocities in proximity to the endothelium might be the most important factor for the elevated LDL concentration.

## Background

Elucidating the blood flow and the transport of macromolecules in the cardiovascular system is essential in understanding the genesis and progression of atherosclerosis [1,2]. Wall Shear Stress (WSS) may affect the endothelial permeability [3,4]. Regional variations in the permeability of arterial endothelium may contribute to the localization of atherosclerosis [5]. The transportation of Low-Density Lipoproteins (LDL) across the artery wall is considered to be a step of paramount importance in atherosclerosis [6,7]. Atherosclerosis shows a predilection in regions of the arterial tree with hemodynamic particularities, such as local disturbances of WSS in space, and locally high concentrations of lipoprotein [8,9] and [10]. In proximal Left Coronary Artery (LCA) tree regions, where atherosclerosis frequently occurs, low WSS appears [9]. The local velocity, the molecular viscosity disturbances and the morphological (geometrical) particularities may also predispose to the formation of coronary atheromatic plaques [11]. Although the WSS has been widely proved to affect the arterial segmental biology, the near-wall localization of critical macromolecular blood particles may significantly contribute to the development of atherosclerotic plaques [12]. LDL is one of these particles, placed there by transport and diffusion.

The strategy and some of the pros and cons of computational modeling approach of the coupled fluid and mass flow (focusing on results from studies made by others) on a variety of arterial geometries have been reviewed by Ethier [1]. He concluded that elevated LDL co-localize with known sites of atherosclerotic plaque development. Besides atherosclerotic regions are low WSS regions. Consequently, it was difficult to determine whether it was low WSS, abnormal mass transfer or both that were contributing to astherosclerosis. A theoretical study of a straight artery by Wada et al. suggested the possibility that all vascular phenomena were governed by the flow-dependent concentration polarization of LDL, which carries cholesterol [13]. A multiple bend was studied to elucidate the mechanisms of localization of atherosclerotic lesions [14]. They drove into the conclusion that regions of elevated LDL luminal surface concentration did not necessarily co-located to the sites of lowest WSS. They used constant value for the blood molecular viscosity, instead of a non-linear one [15].

The accumulation of LDL in vascular districts featuring highly disturbed flow was examined [16]. Geometrical parameters such as curvature and variations of the luminal section strongly influence the LDL within the wall. Realizing that the research in macromolecular transport of curved 3D blood vessels was rare, steady and unsteady flow and mass simulation was analyzed [17]. However, the blood was treated as a Newtonian fluid, thus limiting the quantitative results. They concluded that the concentration of LDL along the aortic axis yields higher values at the outer wall (concave side) compared to inner wall (convex side).

The current study is a three-dimensional, numerical simulation that couples the flow equations with the transport equation, applying realistic boundary conditions, in terms of blood-side concentration at the wall. The blood is considered to be non-Newtonian fluid obeying to the power law. Emphasis has been put on: a) LDL (concentration) distribution, b) factors affecting mass transport from flowing blood to arterial wall using various water infiltration velocity, and molecular diffusivity, c) WSS distribution. We demonstrate that due to the semi-permeable nature of the arterial walls, high concentrations of LDL occur at the endothelium. The LDL variation is most noticeable at bifurcations opposite to the flow dividers and at the concave parts of the bent arterial segments.

## Methods

### Geometry and computational grid

**Figure 1:**
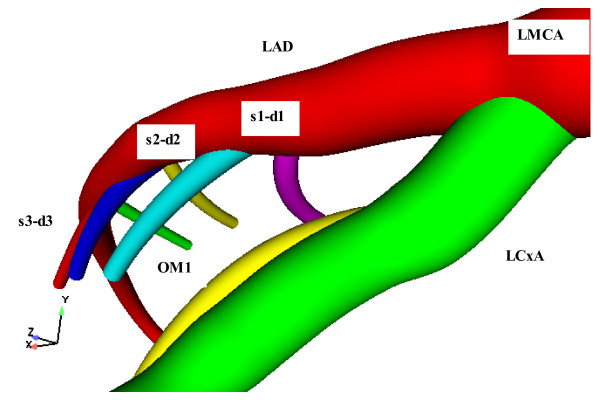
**LCA Model**. Normal LCA tree geometry. Includes LMCA, LAD and LCxA.

**Figure 2:**
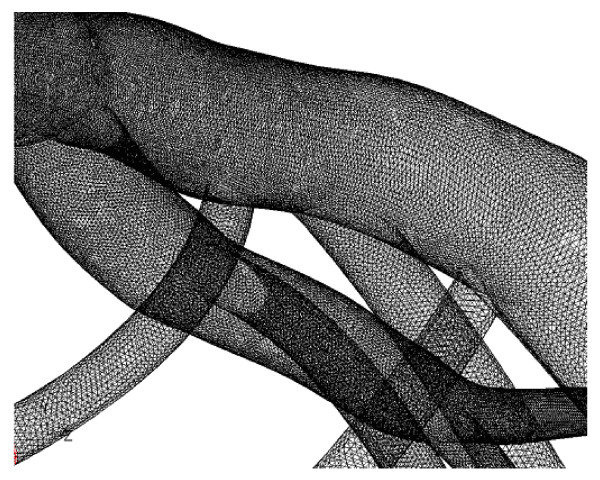
**Mesh**. Non-structured grid of the normal human LCA tree is used for the computational analysis. Details of proximal segments are shown.

Experimental measurements of the intrathoracic spatial location of specified coronary segments on the normal human heart have been previously reported [18,19] The intrathoracic location and course of each one of the 23 arterial segments and branches, which are commonly used by the physician to describe the localization of coronary disease, were processed with a CAD program. This programme produced a three-dimensional geometry model of the LCA tree, Fig. 1. Angiographic data used for geometry construction correspond to end-diastole. The resulted model included the LMCA, LAD, LCxA and their main branches. All geometrical data has been processed by a specialized pre-processing program for grid generation. In total 173404 grid nodes were utilized giving rise to 499113 computational tetrahedral. Figure 2 shows some details of the utilized non-structured grid of the human LCA tree.

### Flow equations and assumptions

All computational grid data as well as all physical flow data determined from the boundary conditions were imported into the main Computational Fluid Dynamics solver [20]. The numerical code solves the governing Navier-Stokes flow equations and the mass transport of LDL equation in a coupled way [21]. The assumptions made about the nature of the flow are that it is three-dimensional, steady, laminar, isothermal, with no external forces applied on it. The arterial wall is comprised from non-elastic and permeable material. In their generality the governing flow equations are,

(1) $$\frac{\partial \rho }{\partial t}+\nabla •\left(\rho \vec{u}\right)=0$$

*ρ *(kg/m^3^) is the density, *t *(sec) is the time, $\vec{u}$ (m/s) is the velocity vector. The conservation of momentum is written,

(2) $$\frac{\partial }{\partial t}\left(\rho \vec{u}\right)+\nabla •\left(\rho \vec{u}\vec{u}\right)+\nabla p=\nabla •(\bar{\tau })+\rho \vec{g}$$

Here, *p *(N/m^2^) is the static pressure, $\bar{\tau }$ (N/m^2^) is the shear stress tensor and *ρ*$\vec{g}$ (N/m^3^) is the gravitational body force. The shear stress tensor $\bar{\tau }$ is given by,

(3) $$\vec{\tau }=\mu \left[\left(\nabla \vec{u}+\nabla {\vec{u}}^{T}\right)\right]-\frac{2}{3}\nabla •\vec{u}I.$$

*μ *is the molecular viscosity, *I *is the unit tensor, and the second term in the right hand side is the effect of the volume dilation.

The blood was considered to be non-Newtonian fluid obeying to the power law [22]. According to this law the molecular viscosity, now denoted as *η*($\dot{S}$), is given by,

(4) $$\eta \left(\dot{S}\right)=ke^{\frac{T_{o}}{T}}{\dot{S}}^{n-1}$$

$\dot{S}$ is the shear rate given by,

(5) $$\dot{S}=\frac{\partial u_{i}}{\partial x_{j}}+\frac{\partial u_{j}}{\partial x_{i}}$$

The consistency index *k *is 0.01691 (kg-s^n-2/m), the power-law index *n *is 0.7, *T*(K) and *T*_*o*_(K) are local and reference temperatures, respectively. The components of the WSS possibly have different effects upon endothelial cells. Some components, mainly those being diagonal, generate intercellular tension while the off-diagonal components possibly contribute to intercellular shearing forces [23]. Thus, the actual shear stress is given by,

(6) $$\tau =\left[\eta \left(\dot{S}\right)\right]\dot{S}$$

The solution of the convection-diffusion equation is achieved by,

(7) $$\frac{\partial (\rho C)}{\partial t}+\nabla •\left(\rho \vec{u}C\right)+\nabla •\vec{J}=0$$

*C *(mg/ml) is the LDL concentration, $\vec{J}$ is the diffusion flux of LDL, which arises due to concentration gradients. The diffusion flux is written as,

(8) $$\vec{J}=-\rho D\nabla C$$

*D *(m^2^/s) is the diffusion coefficient of LDL in the mixture.

### Blood diffusivity, water infiltration velocity and endothelial permeability

In the absence of systematic and reliable experimental data for human arteries, we assume the molecular diffusivity D to be isotropic and constant [13,24]. Various molecular diffusivities and water infiltration velocities V_w _are applied to calculate the LDL luminal surface concentration over the LCA tree. There is a limited amount of experimental data about the endothelial permeability K (mass transfer coefficient). Thus, specifications of K values are sometimes unreliable. In the current analysis the K value is set equal to 2.0 × 10^-10 ^m/s [14].

### Flow boundary conditions

The blood velocity is assumed to be uniform at the orifice of LMCA. The applied inflow conditions mimic typical coronary blood averaged flow velocities of 0.05 m/s under resting conditions, corresponding to Reynolds number of 95. The blood density is set equal to 1058 kg/m^3^. Flow discharges are set analogous to the third power of the branching vessel inlet diameter according to Murray's law [25].

### Mass transport boundary conditions

For the mass transport solution Eq. (7), a uniform constant concentration *C*_*o *_of LDL (= 1.3 mg/ml) is applied at the coronary tree inlet. At the coronary artery vessel outlets, the gradient of LDL concentration along the vessel is set equal to zero (zero flux, Newmann condition) $\frac{\partial C}{\partial s}=0$, *s *is the unit vector normal to the outlet surfaces of the LCA. Suitable mass transport condition must be specified at the wall. The boundary conditions at the wall can be described as,

(9) $$C_{w}V_{w}-D\frac{\partial C}{\partial n}=KC_{w}$$

*C*_*w *_(mg/ml) is the concentration at the endothelial surface, *V*_*w *_(m/s) is the transmural component of the fluid velocity at the wall, *n *is the direction normal to the wall. The boundary conditions described in Eq. (9) state that the net amount of LDL (= *K C*_*w*_) passing from the endothelium to the vessel wall is determined from the difference of the mass flow carried to the vessel wall by infiltration flow (= *C*_*w*_*V*_*w*_) and the amount of flow which diffuses back to the main vessel flow (= $D\frac{\partial C}{\partial n}$). It is known that the concentration of LDL at an arterial wall is higher than that in the bulk flow and it increases with increasing infiltration velocity [13].

### Solution

The user defined function capabilities of the numerical code are also incorporated to simulate the mass transport boundary condition across the endothelium, Eq. (9). For a typical satisfactory convergence solution, a total of 1000 pseudo-time steps are required. Convergence is achieved when all velocity components, fluid flow, and mass flow changes dropped below 10^-8^.

## Results

### WSS and luminal surface LDL concentration at the LMCA bifurcation. LDL versus WSS over the entire LCA tree

All LDL luminal surface concentration values Cw are normalized with the inlet value of LDL concentration Co (= 1.3 mg/ml). The applied flow conditions refer to inlet based Reynolds number Re_o _= 95 using V_w _= 0.6 × 10^-8 ^m/s, uniform constant LDL concentration, endothelial permeability K = 2.0 × 10^-10 ^m/s and diffusivity D = 15.0 × 10^-12 ^m^2^/s.

**Figure 3:**
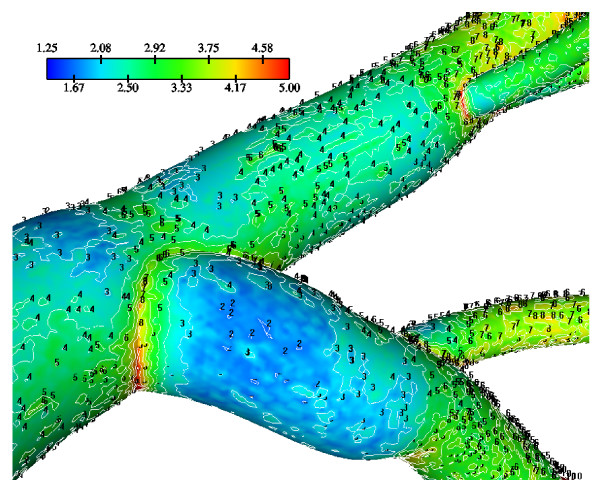
**WSS Contours**. WSS (N/m^2^) magnitude contour plots of the Left Main Coronary Artery (LMCA) bifurcation. Low WSS regions occur opposite to the flow dividers. These anatomic sites are predisposed for atherosclerotic development.

**Figure 4:**
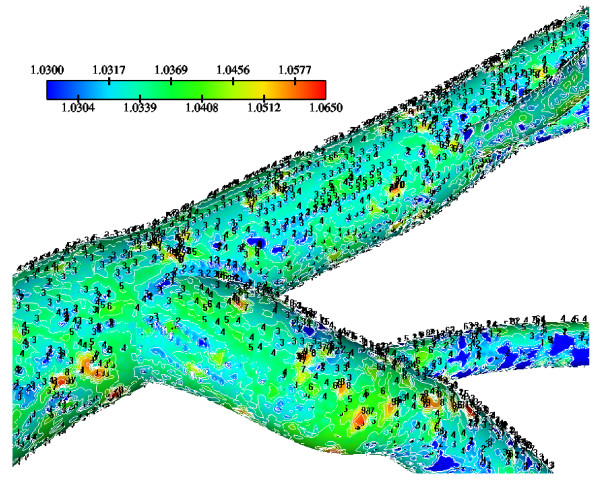
**LDL at bifurcation**. Normalized luminal surface LDL concentration of the LMCA bifurcation. High LDL regions occur opposite to the flow dividers.

The WSS results indicate that in the LMCA bifurcation at regions opposite to the flow divider dominant low values occurs ranging from 1.67 N/m^2 ^to 2.50 N/m^2^, Fig. 3. High curvature affects the velocity distribution at the flow divider, giving rise to high WSS values. The normalized LDL luminal surface concentration values (= Cw/Co) are shown in Fig. 4. Elevated LDL values appear at regions opposite to the flow divider at either LMCA, or proximal LAD or LCxA branches. The spatial gradients of the LDL are considerable. The results indicate that at the main tree bifurcation the normalized LDL luminal surface concentration values range between 1.0369–1.0577.

For rigorous exercise flow conditions of 0.34 m/s, the corresponding Reynolds number is 640. Table 1 shows the area averaged LDL luminal surface concentration of Cw/Co over the LCA tree segments for Re_o _= 640. The area averaged normalized LDL concentration over the entire LCA tree is 1.0347.

**Table 1:** Area averaged LDL concentration Cw/Co over LCA tree segments

LCxA	OM1	OM2	S1	D1	S2	D2	S3	D3	LAD
1.0345	1.0347	1.0338	1.0346	1.0343	1.0350	1.0350	1.0353	1.0361	1.0349

Area averaged LDL concentration Cw/Co over LCA tree segments. Values refer to Re_o _= 640, V_w _= 0.6 × 10^-8^m/s, K = 2.0 × 10^-10 ^m/s and D = 15.0 × 10^-12^m^2^/s. The area averaged LDL of Cw/Co over the entire LCA tree is 1.03475

**Figure 5:**
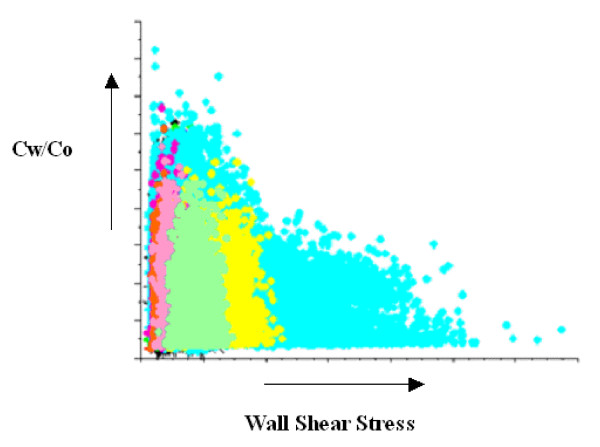
**LDL versus WSS**. Typical luminal surface concentration of normalized LDL versus WSS.

A typical relationship between luminal surface concentrations of the normalized LDL with WSS, over the entire LCA tree surface, is shown in Fig. 5. It is evident that the wall LDL concentration increases with decreasing WSS. When the WSS values are reduced to zero, the rate of LDL increase is getting higher. Although WSS is a factor that determines the endothelial LDL concentration, is not the only one.

### LDL at distal bend surface of the LCA

**Figure 6:**
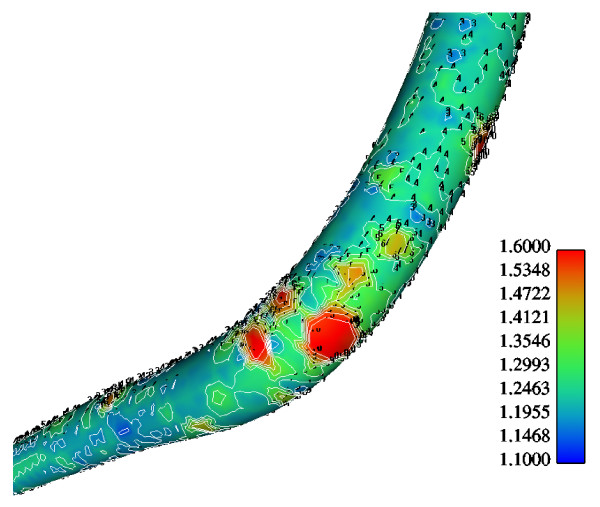
**LDL at the concave side of the distal LAD**. Contour plots of the normalized luminal surface LDL concentration at the concave side of the distal LAD. The flow direction is from up-right to down-left. The concave part of the LAD exhibits, relative to the convex side, high LDL concentration.

**Figure 7:**
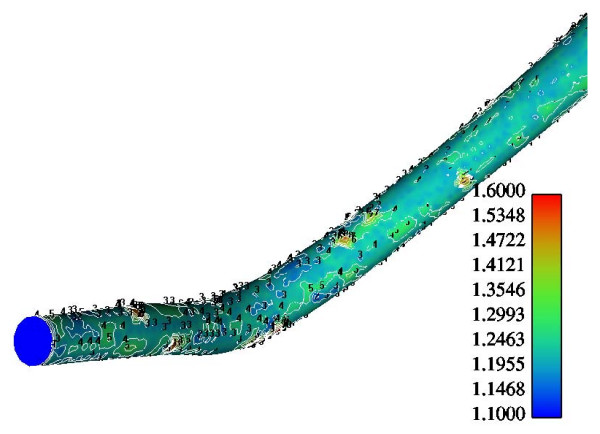
**LDL at the convex side of the distal LAD**. Contour plots of the normalized luminal LDL surface concentration at the convex side of the distal LAD. The flow direction is from up-right to down-left.

**Figure 8:**
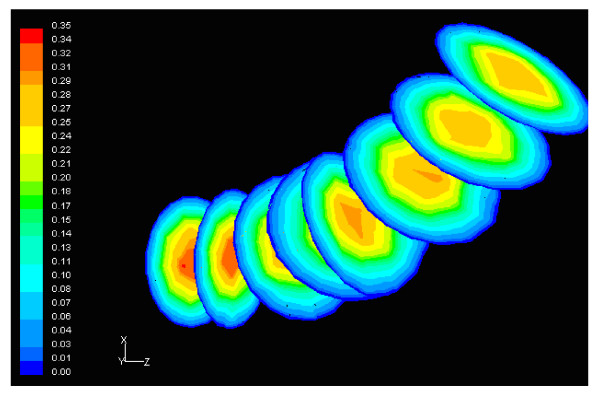
**Velocity**. Contour plots of velocity magnitude (m/s), at various cross-sections of the distal LAD segment shown in Fig. 6. The flow direction is from up-right to down-left.

**Figure 9:**
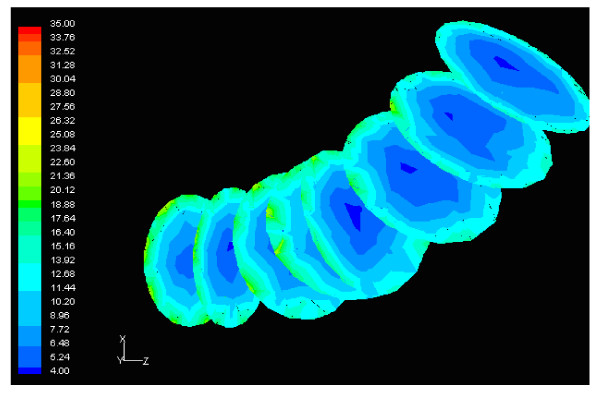
**Strain rate**. Contour plots of strain rate (1/s) at various cross-sections of the distal LAD segment shown in Fig. 6. The flow direction is from up-right to down-left. The strain rate is low at areas of high LDL concentration. Further downstream, as the LDL decreases the strain rate increases.

**Figure 10:**
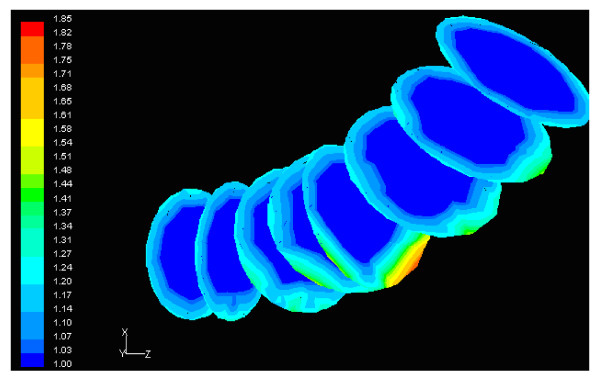
**LDL**. Contour plots of normalized LDL concentration at various cross-sections of the distal LAD segment shown in Fig. 6. The flow direction is from up-right to down-left.

Figure 6 shows the normalized luminal surface LDL concentration at the concave side (outer wall) of the distal LAD segment. The vessel geometry exhibits high bend at this regional area. The normalized peak LDL value is 60.0% higher than that at the entrance. The convex side (inner wall) of the distal LAD segment exhibits relatively to the concave side lower luminal surface LDL concentrations ranging from 19.5% to 29.9% higher than that at the entrance, Fig. 7. The contour plots of velocity magnitude (m/s), strain rate (velocity change with distance) and normalized LDL concentration at various distal LAD cross-sections are shown in Figs. 8, 9 and 10, respectively. Elevated LDL concentration is located at concave parts of the bend, Fig. 10. These regions exhibit low strain rates, Fig. 9.

### LMCA bifurcation. Water infiltration effect in luminal surface LDL concentration

Various water infiltration velocities V_w _are applied to calculate the LDL luminal surface concentration and WSS over the LCA tree. An inlet flow velocity of 0.05 m/s (Re_o _= 95) is applied to simulate the fluid flow-mass transport problem using endothelial permeability K = 2.0 × 10^-10 ^m/s, and molecular diffusivity D = 15.0 × 10^-12 ^m^2^/s. The applied infiltration velocities are 0.6 × 10^-8 ^m/s (low), 1.0 × 10^-8 ^m/s (median), and 4.0 × 10^-8^m/s (high).

**Figure 11:**
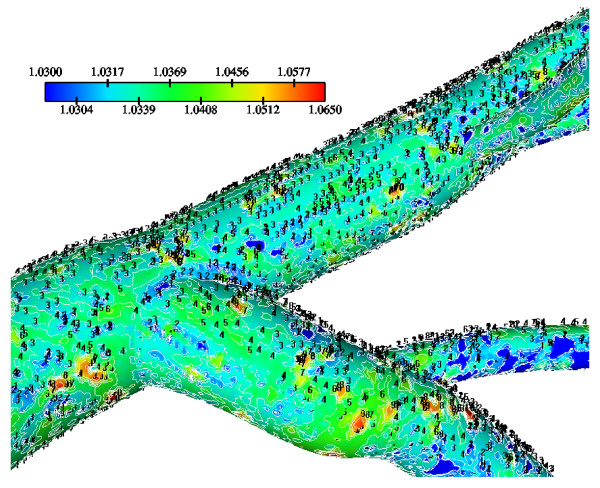
**LDL contours using various V_w _= 0.6 × 10^-8^m/s**. Water infiltration velocity effects of the normalized luminal surface LDL concentration in the LMCA bifurcation at Re_o _= 95, K = 2.0 × 10^-10 ^m/s and D = 15.0 × 10^-12 ^m^2^/s with V_w _= 0.6 × 10^-8 ^m/s.

**Figure 12:**
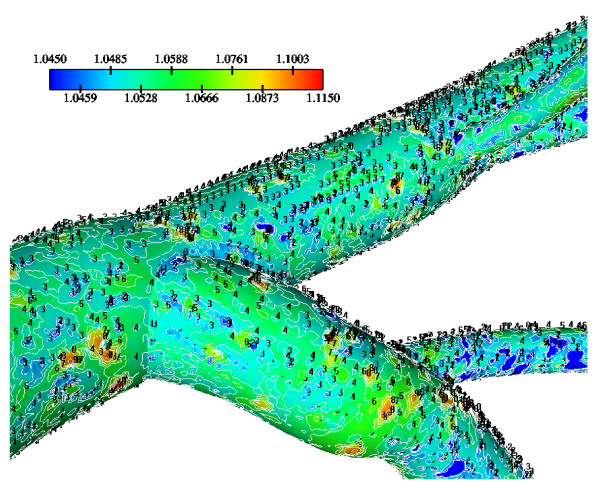
**LDL contours using various V_w _= 1.0 × 10^-8^m/s**. Water infiltration velocity effects of the normalized luminal surface LDL concentration in the LMCA bifurcation at Re_o _= 95, K = 2.0 × 10^-10 ^m/s and D = 15.0 × 10^-12 ^m^2^/s with V_w _= 1.0 × 10^-8 ^m/s.

**Figure 13:**
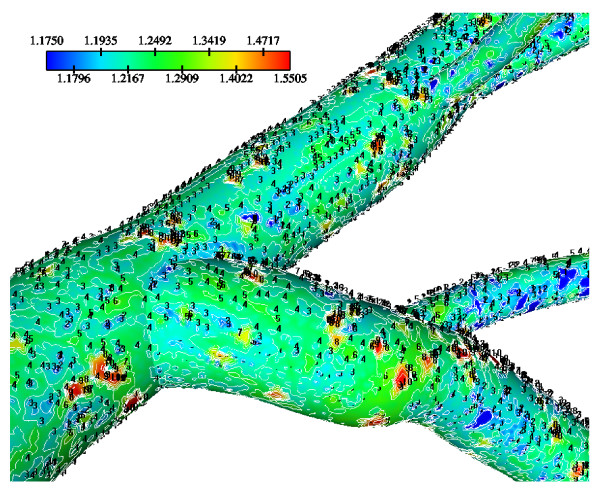
**LDL contours using various V_w _= 4.0 × 10^-8^m/s**. Water infiltration velocity effects of the normalized luminal surface LDL concentration in the LMCA bifurcation at Re_o _= 95, K = 2.0 × 10^-10 ^m/s and D = 15.0 × 10^-12 ^m^2^/s with V_w _= 4.0 × 10^-8 ^m/s.

Calculated WSS differences using various water infiltration velocities, particularly at regions opposite to flow dividers are very small (not shown). Normalized luminal surface LDL concentrations at low, median and high infiltration velocity values are shown in Figs. 11, 12 and 13, respectively. High infiltration velocity results into elevated luminal surface LDL concentration. High concentration values occur at regions opposite to the flow divider at either LMCA, or proximal LAD or LCxA branches. For high, median and low infiltration velocities the peak normalized LDL luminal surface concentrations at the LMCA bifurcation are 1.550, 1.115 and 1.065, respectively.

Tables 2, 3 and 4 show the area averaged normalized LDL concentration values for the low, median and high infiltration velocities, respectively, over the LCA tree segments. Area-averaged normalized LDL concentrations over the entire LCA tree are 3.5%, 5.4% and 23.2% higher than that at the entrance corresponding to the low, median and high water infiltration velocities, respectively. The net amount of LDL mass per second taken up by all LCA tree luminal surfaces is 2.54 × 10^-11 ^g/s, 4.32 × 10^-11 ^g/s and 20.17 × 10^-11 ^g/s for the low, median and high infiltration velocities, respectively.

**Table 2:** Water velocity filtration effects at V_w _= 0.6 × 10^-8 ^m/s

LCxA	OM1	OM2	S1	D1	S2	D2	S3	D3	LAD
1.0345	1.0346	1.0339	1.0347	1.0344	1.0347	1.0350	1.0352	1.0361	1.0350

Water velocity filtration effects. Area averaged LDL concentration Cw/Co. Values refer to Re_o _= 95, K = 2.0 × 10^-10 ^m/s and D = 15.0 × 10^-12 ^m^2^/s, V_w _= 0.6 × 10^-8 ^m/s. The area averaged LDL concentration Cw/Co over the entire LCA tree is 1.0348. The net amount of LDL mass per second taken up by all LCA tree luminal surface is 2.54 × 10^-11^g/s.

**Table 3:** Water velocity filtration effects at V_w _= 1.0 × 10^-8 ^m/s

LCxA	OM1	OM2	S1	D1	S2	D2	S3	D3	LAD
1.0548	1.0542	1.0528	1.0540	1.0535	1.0546	1.0547	1.0551	1.0567	1.0548

Water velocity filtration effects. Area averaged LDL concentration Cw/Co. Values refer to Re_o _= 95, K = 2.0 × 10^-10 ^m/s and D = 15.0 × 10^-12 ^m^2^/s, V_w _= 1.0 × 10^-8 ^m/s. The area averaged LDL concentration Cw/Co over the entire LCA tree is 1.0542. The net amount of LDL mass per second taken up by all LCA tree luminal surface is 4.32 × 10^-11 ^g/s.

**Table 4:** Water velocity filtration effects at V_w _= 4.0 × 10^-8 ^m/s

LCxA	OM1	OM2	S1	D1	S2	D2	S3	D3	LAD
1.2352	1.2311	1.2219	1.2280	1.2277	1.2314	1.2340	1.2344	1.2460	1.2352

Water velocity filtration effects. Area averaged LDL concentration Cw/Co. Values refer to Re_o _= 95, K = 2.0 × 10^-10 ^m/s and D = 15.0 × 10^-12 ^m^2^/s, V_w _= 4.0 × 10^-8 ^m/s. The area averaged LDL concentration Cw/Co over the entire LCA tree is 1.2325. The net amount of LDL mass per second taken up by all LCA tree luminal surface is 20.17 × 10^-11^g/s.

### LMCA bifurcation. Molecular diffusivity effect in luminal surface LDL concentration

**Figure 14:**
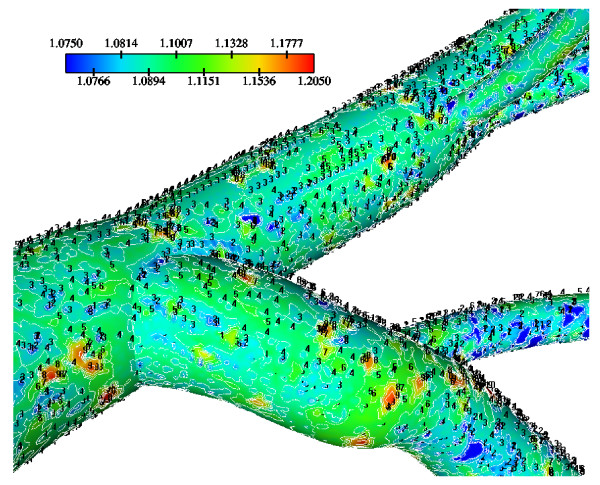
**LDL contours using various D = 5.0 × 10^-12^m^2^/s**. Diffusivity effects on the normalized luminal surface LDL concentration in the LMCA bifurcation at Re_o _= 95, K = 2.0 × 10^-10 ^m/s and V_w _= 0.6 × 10^-8 ^m/s with D = 5.0 × 10^-12^m^2^/s.

**Figure 15:**
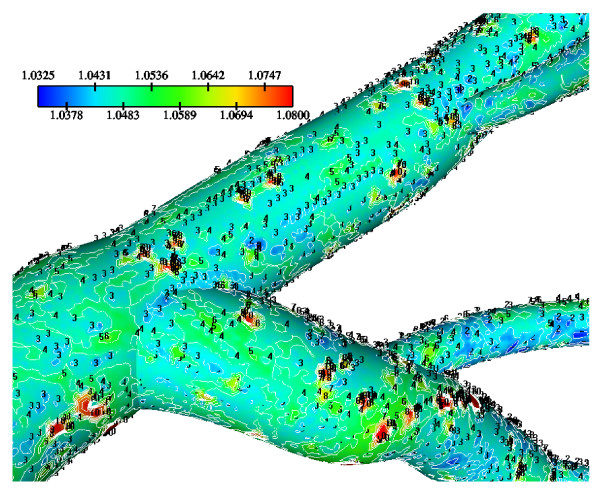
**LDL contours using various D = 10.0 × 10^-12^m^2^/s**. Diffusivity effects on the normalized luminal surface LDL concentration in the LMCA bifurcation at Re_o _= 95, K = 2.0 × 10^-10 ^m/s and V_w _= 0.6 × 10^-8 ^m/s with D = 10.0 × 10^-12^m^2^/s.

**Figure 16:**
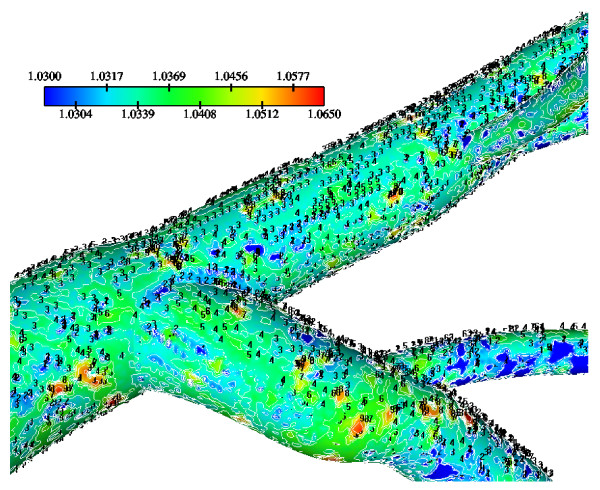
**LDL contours using various D = 15.0 × 10^-12^m^2^/s**. Diffusivity effects on the normalized luminal surface LDL concentration in the LMCA bifurcation at Re_o _= 95, K = 2.0 × 10^-10 ^m/s and V_w _= 0.6 × 10^-8 ^m/s with D = 15.0 × 10^-12^m^2^/s.

Molecular diffusivity D values using 5.0 × 10^-12 ^m^2^/s (low), 10.0 × 10^-12 ^m^2^/s (median) and 15.0 × 10^-12 ^m^2^/s (high) at Re_o _= 95, K = 2.0 × 10^-10 ^m/s and V_w _= 0.6 × 10^-8 ^m/s are applied. The normalized luminal surface LDL concentration for low, median and high molecular diffusivity values are shown in Figs. 14, 15 and 16, respectively. High molecular diffusivity results into low LDL concentration for all LCA tree segments. Furthermore, low molecular diffusivity results into increased area of elevated LDL throughout the LCA tree. High concentration values occur at regions opposite to the flow dividers. For the high, median and low molecular diffusivities, the peak normalized LDL luminal surface concentrations at the LMCA bifurcation reach 1.065, 1.080 and 1.205, respectively. The effect of molecular diffusivity upon the values and the patterns of WSS distribution are marginal (not shown).

Tables 5, 6 and 7 show the area averaged normalized LDL concentration values for the low, median and high molecular diffusivities, repectively, over the LCA tree segments. Area-averaged normalized LDL concentrations over the entire LCA tree are, 9.4%, 4.9% and 3.5% for the low, median and high molecular diffusivity, respectively. The net amount of LDL mass per second taken up by all LCA tree luminal surfaces is 2.68 × 10^-11^g/s, 2.57 × 10^-11^g/s and 2.54 × 10^-11^g/s corresponding to the low, median and high molecular diffusivities, respectively.

**Table 5:** Molecular diffusivity effects at D = 5.0 × 10^-12 ^m^2^/s

LCxA	OM1	OM2	S1	D1	S2	D2	S3	D3	LAD
1.0918	1.0932	1.0909	1.0932	1.0925	1.0940	1.0947	1.0950	1.0985	1.0947

Molecular diffusivity effects. Area averaged LDL concentration Cw/Co. Values refer to Re_o _= 95, K = 2.0 × 10^-10 ^m/s and V_w _= 0.6 × 10^-8 ^m/s, D = 5.0 × 10^-12 ^m^2^/s. The area averaged LDL concentration Cw/Co over the entire LCA tree is 1.0939. The net amount of LDL mass per second taken up by all LCA tree luminal surface is 2.68 × 10^-11^g/s.

**Table 6:** Molecular diffusivity effects at D = 10.0 × 10^-12 ^m^2^/s

LCxA	OM1	OM2	S1	D1	S2	D2	S3	D3	LAD
1.0479	1.0486	1.0475	1.0487	1.0482	1.0493	1.0493	1.0495	1.0510	1.0493

Molecular diffusivity effects. Area averaged LDL concentration Cw/Co. Values refer to Re_o _= 95, K = 2.0 × 10^-10 ^m/s and V_w _= 0.6 × 10^-8 ^m/s, D = 10.0 × 10^-12 ^m^2^/s. The area averaged LDL concentration Cw/Co value over the entire LCA tree is 1.0489. The net amount of LDL mass per second taken up by all LCA tree luminal surface is 2.57 × 10^-11 ^g/s.

**Table 7:** Molecular diffusivity effects at D = 15.0 × 10^-12 ^m^2^/s

LCxA	OM1	OM2	S1	D1	S2	D2	S3	D3	LAD
1.0342	1.0346	1.0339	1.0347	1.0344	1.0347	1.0350	1.0352	1.0361	1.0350

Molecular diffusivity effects. Area averaged LDL concentration Cw/Co. Values refer to Re_o _= 95, K = 2.0 × 10^-10 ^m/s and V_w _= 0.6 × 10^-8 ^m/s, D = 15.0 × 10^-12 ^m^2^/s. The area averaged LDL concentration Cw/Co value over the entire LCA tree is 1.0348. The net amount of LDL mass per second taken up by all LCA tree luminal surface is 2.54 × 10^-11^g/s. The Cw/Co values over the LCA tree segments are.

## Discussion

### Mass transfer importance

Main purpose of this computational study is to elucidate the LDL site concentration under various mass conditions. The arising are the degree of elevation of the luminal surface LDL concentration and the area of the LCA tree that it occupies in relation to water infiltration and size of macromolecules. The amount of LDL transported to the arterial wall is a function of the surface LDL elevation. However, the transported LDL is related to the permeability of the arterial wall, which in turn is a function of the WSS. The blood and the arterial wall constantly exchange substances. In normal flow conditions the mass exchange between blood flow and the near to arterial wall material is kept constant. Wherever and whenever the flow becomes abnormal, the balance between the mass flow of substances from the main blood stream to the arterial wall and vice versa is disrupted [26]. In this case the unbalanced mass transfer probably initiates the arterial wall disease. The mass transfer quantification is crucial in the genesis and the progression of atherosclerosis. Understanding the flow behaviour at the near arterial wall region is of paramount importance. However, mass transport is a highly complex physical phenomenon depending upon biological, chemical and mechanical factors. The action of mass transport occurs within a very thin layer, which is located very close to the endothelium. Thus, any perturbation of the flow in this sensitive region initiates mass flow disturbance to and from endothelium.

### Wall Shear Stress and endothelium

The endothelium permeability is particularly sensitive to local flow perturbations caused from various mechanical factors. It has been proved that endothelial cells are subject to morphological alterations which are activated via changes of WSS magnitude as well as WSS orientation [3]. Elongation of the endothelial cells occurs in regions of high WSS, with the longest cell axis oriented parallel to the flow direction [27]. Conversely, in low WSS regions, the endothelial cells have polygonal shape with no specific orientation. It is possible that these local alterations in endothelial cell morphology have different degrees of permeability for the various blood constituents. Therefore, endothelial cells from regions of various degrees of WSS exhibit various biological and biochemical functions [3]. Since the WSS interacts with the endothelial cells and subsequently with the arterial wall permeability, it is difficult to determine whether it is low WSS or abnormal mass transfer coefficient (permeability coefficient) or both that contribute to atherosclerosis.

### Wall Shear Stress and Low-Density Lipoprotein concentration

In the LMCA bifurcation at regions opposite to the flow divider, predominantly low WSS values occur. High WSS values occur at the "cervix" of the origin and at the flow divider. At the "hips" of the LMCA bifurcation, i.e. at regions opposite to the flow divider, located at the outer walls of LAD and LCxA, low WSS values occur, Fig. 3. High curvatures affect the velocity distribution at vessel bends giving rise to high WSS values. Furthermore, the WSS exhibits high values in the distal regions of the LCA, where the magnitude of the mean flow velocity is relatively higher, due to vessel tapering.

The relationship between WSS and luminal surface concentration of LDL, shown in Fig. 5, indicates that LDL is elevated at locations where WSS is low. As WSS values approach zero, the LDL concentration rapidly increases. A lot of points in the graph may indicate that the LDL concentration is not only WSS dependent. Other flow parameters may seriously affect the flow pattern. Most research workers try to elucidate atherosclerosis by studying the WSS distribution. According to the prevailing theory, low WSS is responsible for atherosclerosis process. Current research analysis results that the combined fluid and mass transport are the keys to understand atherosclerosis. Under the action of the flow the LDL particles move to reach a particular site. Thereafter, it is the contact time and the interaction between LDL and endothelial surface which really matters. The net amount of LDL passing through the arterial wall depends upon the particular physical properties of the wall material (permeability).

The results indicate that LCA tree walls are exposed to a cholesterolemic environment although the applied mass and flow conditions refer to normal human geometry and mass-flow conditions. High luminal surface concentration does not necessarily denote that all the LDL molecules will be transported through the vessel wall. It is the permeability of the endothelium, which mainly determines the final amount passing through the wall.

The results also demonstrate that regions of low WSS values do not necessarily coincide with elevated LDL concentration regions. Low WSS regions exhibit elevated LDL concentrations. At the LMCA bifurcation, the peak LDL concentration is identified just upstream of the low WSS area (regions opposite the flow divider, located at the outer walls of LAD and LCxA) or just downstream of it, Figs. 3 and 4. Concave parts exhibit elevated LDL concentration. On the other hand, convex parts exhibit low LDL concentrations. For the distal LAD vessel the mechanism of elevated LDL concentration might be also attributed to high curvature effects upon the flow pattern and the subsequent spatial accumulation of the LDL. This is clearly shown in strain rate contours, Fig. 9. The paths of the velocities in proximity to the endothelium might be the most important factor for the elevated LDL concentration in either of these two high curvature areas, i.e. areas located either at the vicinity of bifurcations regions or at high curvature regions. At near wall areas, low strain rates occur at regions where elevated LDL concentration is present, Figs 8, 9 and 10. Low strain rates located near to endothelium cover larger areas than the corresponding convex part. Further downstream, increased strain rates are present at the near to wall region, while the distribution of the LDL concentration exhibits low values. The importance of fluid flow and mass transport at the near wall regions becomes apparent. Similar conclusions are reported testing a high curvature bend of RCA with flow separation [14].

As the water infiltration velocity increases, the luminal surface concentration of LDL increases as well. Henceforth, the net amount of LDL mass per second taken up by LCA tree luminal surfaces is also increased. At the low water velocity filtration value, the area averaged LDL concentration over the entire LCA tree is only 3.48% higher than at the inlet. However, at the maximum water infiltration velocity the corresponding value is 23.25%. This indicates that the water infiltration velocity parameter is an important factor for LDL concentration.

High molecular diffusivity results in low LDL concentration on all LCA tree segments. At low molecular diffusivity, the area averaged LDL concentration over the entire LCA tree is 9.39% higher than at the entrance. The concentration attains 4.89% and 3.48% values for the median and maximum molecular diffusivity, respectively. As the blood diffusivity increases, more LDL masses pass from endothelium to the main flow, reducing the LDL concentration at vessel walls.

The assumption that the permeability of the wall to LDL is constant throughout the LCA tree needs to be reconsidered. A new approach will consider the permeability of the wall to be a function of either the WSS or spatial gradient of WSS. Coupled luminal blood flow and transmural fluid flow will be a natural extension of the current research work. Furthermore, arterial wall deformation needs to be taken into consideration, since the flow pattern-mass transport is largely determined by the geometrical configuration of the artery. Arterial movement has to be taken into account since the applied acceleration force will yield a different flow field. It is expected that the flow pattern will be seriously altered, particularly at distal LCA tree regions.

## Conclusion

The present study predicts increased permeation of LDL concentration at specific regions. WSS plays an important role in the wall concentration of the LDL. At low WSS regions, which appear at bifurcations opposite to the flow dividers, the LDL is elevated. Concave sides of the LCA tree exhibit elevated concentration of the LDL in comparison to the convex sides. The LCA tree walls are exposed to cholesterolemic environment although the applied mass and flow conditions refer to normal human geometry and normal mass-flow conditions. With increasing water infiltration velocity the regional area of high luminal surface concentration is increased. The normalized area-averaged concentrations of LDL over the entire LCA tree are, 3.48%, 5.4% and 23.2% higher than that at the entrance, for the low, median and high water infiltration velocities, respectively. Decreased molecular diffusivity increases the LDL concentration. The normalized area-averaged concentrations of LDL over the entire LCA tree are, 9.39%, 4.89% and 3.48% higher than that at the entrance, for the low, median and high water molecular diffusivities, respectively. The degree of increase in luminal surface concentration of LDL is mostly affected from the water infiltration velocity at the vessel wall. Regions of high LDL luminal surface concentration do not always co-locate to the sites of lowest WSS. The paths of the velocity in proximity to the endothelium might be the most important factor for the elevated LDL concentration at areas located either at the vicinity of bifurcations regions or at high curvature regions.

## Competing interests

The authors declare that they have no competing interests.

## Authors' contributions

Guarantor of integrity of the entire study: JVS. JVS participated in the design of the study and performed the computational analysis. GDG performed analysis results from medical point of view. VCP participated in the computational runs. GEL conceived of the study, and participated in its design and coordination. GEP conceived of the study. All authors read and approved the final manuscript.
